# Social and ethnic disparities in stillbirth and infant death in Denmark, 2005–2016

**DOI:** 10.1038/s41598-021-87084-3

**Published:** 2021-04-12

**Authors:** Trine Damsted Rasmussen, Sarah Fredsted Villadsen, Per Kragh Andersen, Signe Smith Jervelund, Anne-Marie Nybo Andersen

**Affiliations:** 1 Section of Epidemiology, Department of Public Health, University of Copenhagen, Øster Farimagsgade 5, Postbox 2099, 1014 Copenhagen K, Denmark; 2 Section of Social Medicine, Department of Public Health, University of Copenhagen, Øster Farimagsgade 5, Postbox 2099, 1014 Copenhagen K, Denmark; 3 Section of Biostatistics, Department of Public Health, University of Copenhagen, Øster Farimagsgade 5, Postbox 2099, 1014 Copenhagen K, Denmark; 4 Department of Public Health, Section for Health Services Research, University of Copenhagen, Øster Farimagsgade 5, Postbox 2099, 1014 Copenhagen K, Denmark

**Keywords:** Health care, Medical research

## Abstract

Ethnic disparity in stillbirth and infant death has been demonstrated in Europe. As the relation between migration and health change over time, this population based register study investigated the recent figures and explored if potential differences could be explained by the well-known educational and income inequalities in stillbirth and infant death using a novel approach. Stillbirth and infant mortality varied considerably according to country of origin, with only immigrants from China, Norway, and Poland having an overall lower risk than Danish women. Women of Pakistani, Turkish, and Somali origin had a particularly high risk of both outcomes. Women from recent high conflict areas displayed a pattern with increased stillbirth risk. An observed excess risks across generations was found, which is disturbing and rule out factors related to language barriers or newness. Differences in educational level and household income explained only part of the observed inequalities. Strengthening of the maternity care system to better understand and meet the needs of immigrant women seems needed to mitigate the disparities.

## Introduction

Research from Europe, North America, and Australia has reported disparities in stillbirth and infant mortality among ethnic minority groups compared with the majority population^1–7^. A Danish register study documented an increased risk of stillbirth and infant mortality among children born to women from Turkey, Pakistan, and Somalia compared with children born to women of Danish origin, while no differences were observed for immigrants from former Yugoslavia and Lebanon^8^.

In Denmark, the proportion of childbirths among immigrants increased from 11% in 1997 to 19% in 2017^9^. Childbirths among descendants of immigrants are likewise increasing and mainly constituted of women with Turkish and Pakistani origin, being descendants from labour immigrants coming to Denmark in the 1960s and later family reunifications^9^. This group amounted to 3.5% in 2017 and is now sufficient in numbers to study stillbirth and infant death.

Mechanisms explaining the observed differences in stillbirth and infant mortality between ethnic minority populations are complex and not fully determined. Maternal demographics, medical and health behaviour, consanguinity, suboptimal care and socioeconomic factors have been argued to play a role^1^. A systematic review from 2009 highlights the heterogeneity of immigrants and that potential mechanisms differ according to the categorization of immigrant women^1^.

Low socioeconomic position is a well-established risk factor for stillbirth and infant death^10,11^. Immigrants, especially the group of non-Western immigrants, in Denmark constitute a particular socioeconomically disadvantaged group. In 2016, 55% of non-Western immigrants aged 30–59 years, were in the lowest income quintile compared to 15% among the ethnic Danish population^9^. Thus, low socioeconomic position is likely to contribute to ethnic disparities in stillbirth and infant death in Denmark.

Fetal and infant health outcomes are important indicators of the overall health of a population and the quality of health care services^12,13^. The overall rates of stillbirth and infant death in Denmark are low and access to health care is universal and free of charge. With the increased migration, the investigation of ethnic disparities in stillbirth and infant mortality are important to document as prerequisites for the development of preventive strategies and action.

The aim was to study differences in the risk of stillbirth and infant mortality among offspring of immigrants and their descendants in Denmark compared to women of Danish origin in 2005–2016, and whether the potential risk differences could be explained by differences in socioeconomic factors.

## Methods

### Design and study population

The register-linkage study used data on all births with gestational age ≥ 22 weeks born to women with registered residence in Denmark per January 1st in 2005–2016.

This data from the Danish Medical Birth Registry, including information on live- and stillbirths, were linked to information about maternal country of origin, death within the first year of life, maternal educational level, and household income from other national registries at Statistics Denmark.

Information about maternal country of origin was obtained from the Population Registry at Statistics Denmark, using the information registered in the year of birth or, if this was missing, the year following birth or the years before birth. In total, 1947 births were excluded due to missing information on maternal country of origin.

A priori and based on sample size considerations, we restricted the study population to country groups with more than 2000 children born during the study period (38,586 excluded).

The final study population for analysis consisted of 702,705 singleton and multiple births. The live born children were followed-up for mortality until the 31 December 2017.

### Stillbirth and infant mortality

Stillbirth was defined as a child born with no signs of life at or after 22 weeks of gestation, while infant death was defined as death within the first year of life.

### Maternal country of origin

Overall, we used maternal country of origin as the measure of exposure. Based on the definitions by Statistics Denmark^9^, immigrants were defined as being born abroad and having no parents who were born in Denmark and Danish citizens. Descendants were defined as being born in Denmark and having no parents who were born in Denmark and Danish citizens^9^. Women of Danish origin were defined by having at least one parent who was born in Denmark and had a Danish citizenship regardless of the woman’s place of birth^9^. Women of Danish origin constituted the reference group.

### Covariates

Information about year of birth (2005–2008, 2009–2012, 2013–2016), maternal age at delivery (≤ 24, 25–29, 30–34, ≥ 35), maternal parity (1, 2, ≥ 3), multiple births (singleton, twins or more), gestational age at birth (preterm, term, post-term) and birth weight (mean) was drawn from the Danish Medical Birth Registry. Paternal origin was categorized as Danish, immigrant, and descendant.

Maternal educational level was defined as the highest educational level attained or expected (based on ongoing education) in the year of birth or the year prior to if missing in the year of birth. The variable was grouped into three categories: ≤ 9 years, 10–12 years and > 12 years.

Household income was based on the family equivalent disposable income^14^ the year prior to birth or in the year of birth if missing in the year before and categorized into quartiles based on the income distribution of the study population in each birth year.

### Statistical analysis

We calculated proportions of stillbirth (per 1000 births) and infant mortality (per 1000 livebirths) for all subgroups.

Logistic regression analysis was used to estimate odds ratios with 95% confidence intervals as a measure of relative risks of the association between maternal country of origin and, respectively, stillbirth and infant mortality. Robust standard errors were applied to account for dependency in data due to some women giving birth more than once during the study period. We adjusted for the year of birth of the offspring to account for the time trends in stillbirths and infant deaths, as well as the variation in the composition of the different immigrant groups.

Wald tests were used to explore interaction between maternal country of origin and, respectively, maternal education level and household income on the risk of stillbirth and infant mortality.

To examine the potential influence of socioeconomic factors on the association between country of origin and the risk of respectively stillbirth and infant death, we used direct standardization. Direct standardization was conducted separately for each of the outcomes stillbirth and infant death, by fitting a logistic regression model including the exposure maternal country of origin and the socioeconomic variables household income and maternal education level. Based on this model we predicted the risk of stillbirth or infant death for each observation in the dataset and used the joint distribution of household income and maternal education level among women of Danish origin as the standard and averaged the predicted values of women from all other origins using that distribution. Only births with complete information on household income and maternal education level were included in the standardization analyses.

We evaluated possible bias due to missing information on maternal educational level and income in sensitivity analyses by categorizing those with missing information on maternal education level and household income into I) the worst off categories and II) into the best off categories. In addition, we carried out sensitivity analyses including births where information about maternal country of origin was only recorded in the years following birth.

All analyses were conducted using STATA version 14.

### Ethical approval

The study was approved by the Danish Data Protection Agency through the joint notification of The Faculty of Health and Medical Sciences at The University of Copenhagen, November 14 2018 (Record No. 514-0261/18-3000) and all methods were performed in accordance with the relevant guidelines and regulations. According to Danish legislation, further ethical approval or individual consent is not required for register-based research not including human biological material.

## Results

Characteristics of the study population can be seen in Table 1. In total, 617,505 children were born to women of Danish origin, 77,579 children to mothers who immigrated to Denmark, and 7621 children were born to descendants of immigrants (Turkey n = 5357 and Pakistan n = 2264). Women from Syria and women of Turkish descent had the highest number of births in the youngest age group, whereas immigrant women from Somalia, Germany, Sweden, Thailand, and Iran had the highest number of births in the age group above 35 years. Compared to all other origin groups, women from Somalia had the highest frequency of post-term deliveries with a frequency of almost 10%. Mean birth weights were lowest among children born to women from Pakistan and Vietnam and highest among children born to women from Iceland, Ukraine, and Lithuania. The majority of children born to immigrant women had fathers with an immigrant background, with the exception of children born to women from Germany, Norway, Sweden, Thailand, and the Philippines, where the majority had fathers of Danish origin.

**Table 1:** Number of children born and distribution of selected characteristics according to maternal country of origin and descent.

Number of children born by maternal country of origin/ birth	Denmark n=617,505			Turkey n = 7902	Former Yugoslavia n = 7147	Iraq n = 5879	Poland n=5859	Somalia n = 5371	Lebanon n = 4239	Pakistan n = 3922	Germany n = 3468	Afghanistan n = 3258
Number of children born by country of descent	Denmark n = 617,505	Turkey n = 5357	Pakistan n = 2264									
**Year of birth**												
2005–2008	36.5	22.7	27.6	40.2	32.1	38.6	16.0	39.7	40.2	32.1	27.0	29.7
2009–2012	33.3	33.0	34.3	33.6	33.0	31.5	37.7	29.8	33.6	32.3	35.9	31.2
2013–2016	30.2	44.3	38.2	26.3	34.9	29.9	46.4	30.5	26.2	35.6	37.1	39.1
**Multiple births**												
Singleton	95.7	96.8	97.5	95.6	96.8	96.7	96.3	97.5	96.7	97.8	96.3	96.2
Twins or more	4.3	3.2	2.5	4.4	3.2	3.3	3.8	2.5	3.4	2.2	3.7	3.8
**Maternal age at birth**												
≤ 24	11.4	32.5	15.0	11.9	19.0	23.2	13.1	15.7	21.1	12.0	7.4	17.7
25–29	31.3	44.3	38.3	33.4	38.4	29.6	36.8	24.4	34.7	37.5	22.8	34.2
30–34	36.9	19.3	34.7	33.2	27.9	24.4	35.1	26.2	25.0	32.2	39.2	27.1
≥ 35	20.4	4.0	12.0	21.5	14.7	22.8	15.0	33.8	19.2	18.3	30.6	21.1
**Parity**												
1	45.5	49.1	42.1	24.1	39.9	29.5	53.2	18.9	21.7	29.7	48.4	29.0
2	36.4	33.7	30.8	31.9	34.1	27.4	34.9	16.1	24.0	29.4	33.0	26.0
≥ 3	15.8	15.2	24.8	41.2	23.7	41.2	10.0	63.3	52.4	37.8	14.5	42.0
Missing	2.3	2.0	2.3	2.8	2.3	2.0	1.9	1.7	1.9	3.1	4.1	3.0
**Gestational age at birth**												
≤ 36 + 6	6.9	6.2	7.3	6.4	6.1	6.5	6.4	5.8	6.7	6.3	6.7	5.8
37 + 0 − 41 + 6	87.7	90.6	88.7	89.2	89.0	89.4	89.0	83.4	89.3	90.1	86.2	89.1
≥ 42 + 0	4.1	2.9	2.4	3.4	3.5	2.8	3.6	9.8	2.9	2.2	3.5	3.2
Missing	1.4	0.3	0.6	1.1	1.4	1.3	1.0	1.0	1.1	1.4	3.6	1.9
Mean birthweight in grams (SD)	3483 (600)	3373 (567)	3195 (551)	3414 (583)	3451 (583)	3370 (554)	3421 (572)	3391 (592)	3352 (552)	3236 (570)	3433 (579)	3439 (580)
Missing	2.3	1.8	2.9	2.4	2.5	2.7	2.1	2.7	2.6	3.4	4.6	3.5

**Paternal ethnicity**												
Danish origin	95.8	4.1	6.1	2.4	10.7	2.4	30.0	2.4	3.0	1.6	55.8	1.5
Immigrant	3.6	48.0	45.8	81.4	83.1	96.3	68.5	97.2	94.4	74.2	41.9	97.4
Descendant	0.6	47.9	48.1	16.2	6.2	1.3	1.5	0.4	2.6	24.2	2.3	1.1
Missing*												
**Maternal education (years)**												
≤ 9	11.3	26.8	22.7	46.6	27.4	41.3	8.4	53.7	45.9	30.6	5.2	44.1
10–12	34.2	38.4	32.1	29.3	37.4	30.1	21.4	28.7	28.2	27.7	18.4	30.8
>12	53.8	31.0	36.4	12.9	23.7	18.5	32.8	8.1	16.2	18.8	51.9	13.1
Non-registered	0.6	3.8	8.8	11.2	11.5	10.1	37.4	9.5	9.7	22.9	2.5	12.0
**House income (percentiles)**												
≤ 25	20.2	46.2	50.5	62.1	48.2	83.4	38.9	89.7	81.6	70.5	26.4	81.5
> 25–50	25.4	31.3	25.5	25.8	28.9	10.5	30.3	8.6	12.6	20.1	22.9	12.1
> 50–75	27.2	15.5	12.2	8.6	14.7	3.6	17.4	1.3	3.7	6.1	20.8	4.5
> 75–100	27.2	7.0	11.8	3.5	8.2	2.5	13.4	0.4	2.1	3.3	29.9	1.9
Missing*												

Number of children born by maternal country of origin	Norway n = 3115	Sweden n = 2900	Syria n = 2835	Romania n = 2796	Thailand n = 2702	Vietnam n = 2560	Philippines n = 2544	China n = 2380	Iceland n = 2349	Ukraine n = 2243	Iran n = 2068	Lithuania n = 2042
**Year of birth**												
2005–2008	34.5	29.2	11.1	9.7	32.6	36.6	23.5	20.6	34.1	13.8	30.2	18.5
2009–2012	34.5	33.3	12.9	25.1	36.2	35.6	33.4	35.8	35.2	35.6	28.5	34.1
2013–2016	31.0	37.5	76.0	65.3	31.2	27.8	43.1	43.6	30.7	50.6	41.3	47.5
**Multiple births**												
Singleton	95.8	96.0	97.1	96.7	97.0	97.3	97.0	98.2	95.9	96.9	95.8	97.2
Twins or more	4.2	4.0	2.9	3.3	3.0	2.7	3.0	1.8	4.1	3.1	4.2	2.8
**Maternal age at birth**												
≤ 24	6.4	6.0	29.8	13.5	9.0	5.9	5.9	6.6	12.1	10.2	6.4	12.0
25–29	31.0	24.4	33.3	40.8	26.0	30.8	32.9	31.0	38.9	46.8	25.7	38.1
30–34	39.4	42.0	23.4	32.3	35.6	39.6	40.4	40.3	33.5	31.1	38.0	33.8
≥ 35	23.2	27.6	13.5	13.4	29.4	23.7	20.9	22.1	15.5	11.7	29.9	16.1
**Parity**												
1	51.0	53.1	31.6	62.7	41.5	37.7	50.1	59.0	42.2	60.7	49.0	54.9
2	33.3	33.8	25.1	30.1	35.3	37.0	30.6	32.6	33.7	30.7	35.7	35.7
≥ 3	13.5	11.4	36.4	5.9	19.6	23.3	15.3	6.3	22.4	6.9	13.4	7.7
Missing	2.1	1.8	6.9	1.3	3.6	2.0	4.0	2.1	2.0	1.7	1.9	1.7
**Gestational age at birth**												
≤ 36 + 6	6.3	5.8	5.1	6.2	7.9	6.7	8.4	5.0	6.2	4.6	7.0	4.5
37 + 0 − 41 + 6	87.8	88.4	86.6	90.6	88.6	90.5	86.1	91.4	88.2	90.0	89.2	90.2
≥ 42 + 0	4.4	4.7	1.8	2.2	1.4	1.6	2.8	2.5	4.2	4.6	2.5	4.2
Missing	1.4	1.1	6.5	1.0	2.1	1.2	2.7	1.1	1.4	0.8	1.3	1.1
Mean birthweight in grams (SD)	3476 (574)	3475 (559)	3342 (537)	3378 (550)	3366 (552)	3252 (506)	3361 (576)	3459 (521)	3569 (603)	3511 (531)	3348 (571)	3551 (564)
Missing	2.3	1.9	8.4	2.0	3.3	2.1	4.0	1.9	2.5	2.3	2.5	1.9

**Paternal ethnicity**												
Danish origin	79.6	76.6	1.1	21.9	82.0	23.0	76.9	35.9	34.9	30.8	22.4	31.5
Immigrant	18.3	21.0	97.6	77.3	16.8	74.9	21.3	63.0	62.7	68.8	75.5	68.2
Descendant	2.1	2.4	1.3	0.8	1.2	2.1	1.8	1.1	2.4	0.4	2.1	0.3
Missing*												
**Maternal education (years)**												
≤ 9	3.2	4.1	37.3	6.2	37.5	35.9	15.8	9.0	6.0	5.4	18.7	5.8
10–12	16.8	18.7	14.1	21.2	27.6	30.0	32.2	15.5	16.9	14.4	26.5	22.6
>12	62.7	57.8	5.7	30.9	10.4	23.1	16.2	49.1	57.9	46.4	42.0	37.1
Non-registered	17.3	19.4	42.9	41.7	24.5	11.0	35.8	26.4	19.2	33.8	12.8	34.5
**House income (percentiles)**												
≤ 25	26.1	23.5	95.1	45.2	38.2	43.3	44.7	38.6	58.7	43.0	55.1	41.4
> 25–50	21.0	17.7	3.3	30.3	30.5	26.7	29.3	23.1	19.5	29.5	17.3	25.1
> 50–75	21.3	19.7	0.7	14.1	19.3	17.6	16.1	14.2	11.7	15.7	12.1	16.3
> 75–100	31.6	39.1	0.9	10.4	12.0	12.4	9.9	24.1	10.1	11.8	15.5	17.2
Missing*												

Births in Denmark 2005–2016.

Frequencies are given in percentages and in rounded estimates.

*Not listed due to observations < 5 in some cells (missing frequencies is between 0.01–6.9%).

**Include all births to women of Danish origin and to subgroups of immigrants and descendants of immigrants with more than in total 2000 births from 2005–2016.

The lowest levels of maternal education and low household income were seen among immigrant women from Turkey, Iraq, Lebanon, Somalia, Syria, and Afghanistan. Information about the highest attained or ongoing education was missing for a substantial number of immigrants (ranging from 2.5 to 42.9%).

### Maternal country of origin and the risk of stillbirth and infant death

Table 2 shows the absolute risks of stillbirth and infant death and adjusted relative risks, measured as odds ratios with 95% confidence intervals, by maternal country of origin. The stillbirth risk among women of Danish origin was 3.9 per 1000 births. We found substantially increased risk of stillbirth among Turkish and Pakistani descendants (ranging from OR: 1.44–2.32) and among immigrant women from Turkey, Iraq, Somalia, Lebanon, Pakistan, Afghanistan, Syria, the Philippines, Ukraine, and Iran with estimates ranging from 1.25 to almost 3. Women from Vietnam had the lowest risk of stillbirth with a 50% lower risk compared to women of Danish origin, whereas women from Pakistan and Somalia had more than a doubled risk.

**Table 2:** Prevalences, absolute risks and odds ratios (OR) of stillbirth and infant death of children born in Denmark 2005–2016 by maternal country of origin.

Maternal country of origin	Total number of children born	Stillbirth			Infant death		
		No.	No. per 1000 births	Adjusted OR (95% CI)^a^	No.	No. per 1000 live births	Adjusted OR (95% CI)^a^
Denmark	617,505	2405	3.9	1.00	2135	3.5	1.00
Trkish descent	5357	29	5.4	1.44 (1.00–2.07)	23	4.3	1.27 (0.81–1.98)
Pakistani descent	2264	20	8.8	2.32 (1.50–3.60)	17	7.6	2.22 (1.28–3.86)
Turkey	7902	45	5.7	1.45 (1.07–1.97)	48	6.1	1.76 (1.28–2.40)
Former Yugoslavia	7147	33	4.6	1.20 (0.84–1.71)	27	3.8	1.10 (0.75–1.61)
Iraq	5879	38	6.5	1.66 (1.19–2.30)	22	3.8	1.08 (0.70–1.68)
Poland	5859	21	3.6	0.96 (0.61–1.51)	15	2.6	0.76 (0.45–1.31)
Somalia	5371	61	11.4	2.93 (2.22–3.85)	34	6.4	1.84 (1.31–2.58)
Lebanon	4239	22	5.2	1.32 (0.85–2.05)	24	5.7	1.63 (1.03–2.60)
Pakistan	3922	33	8.4	2.20 (1.56–3.09)	38	9.8	2.85 (2.05–3.96)
Germany	3468	15	4.3	1.13 (0.66–1.94)	13	3.8	1.10 (0.64–1.90)
Afghanistan	3258	22	6.8	1.77 (1.12–2.79)	15	4.6	1.35 (0.79–2.32)
Norway	3115	9	2.9	0.74 (0.39–1.43)	7	2.3	0.65 (0.31–1.37)
Sweden	2900	9	3.1	0.81 (0.42–1.56)	10	3.5	1.00 (0.54–1.87)
Syria	2835	23	8.1	2.27 (1.48–3.49)	12	4.3	1.26 (0.71–2.23)
Romania	2796	8	2.9	0.79 (0.36–1.72)	12	4.3	1.29 (0.70–2.37)
Thailand	2702	10	3.7	0.96 (0.51–1.78)	*	*	*
Vietnam	2560	5	2.0	0.50 (0.21–1.20)	11	4.3	1.24 (0.69–2.24)
Philippines	2544	12	4.7	1.25 (0.71–2.21)	10	3.9	1.16 (0.62–2.16)
China	2380	8	3.4	0.89 (0.45–1.79)	7	3.0	0.87 (0.42–1.82)
Iceland	2349	9	3.8	0.99 (0.51–1.90)	9	3.8	1.11 (0.54–2.30)
Ukraine	2243	12	5.3	1.45 (0.82–2.56)	*	*	*
Iran	2068	15	7.3	1.91 (1.15–3.16)	7	3.4	0.99 (0.47–2.07)
Lithuania	2042	8	3.9	1.05 (0.52–2.10)	*	*	*

^a^Adjusted for year of birth.

*Data not shown due to few observations.

**Include all births to women of Danish origin and to subgroups of immigrants and descendants of immigrants with more than in total 2000 births from 2005–2016.

The overall infant death risk in children born to mothers of Danish origin was 3.5 per 1000 live born children. For infant deaths, estimates adjusted for year of birth showed a notable increased risk among immigrant women from Turkey, Somalia, Lebanon, Pakistan, Afghanistan, Syria, and Romania ranging 1.26–2.85 and among women of Turkish and Pakistani descent.

No statistically significant interaction was found between maternal country of origin and, respectively, maternal education level and household income on the risk of stillbirth and infant mortality.

### Are these findings reflections of educational and income disparities?

**Figure 1:**
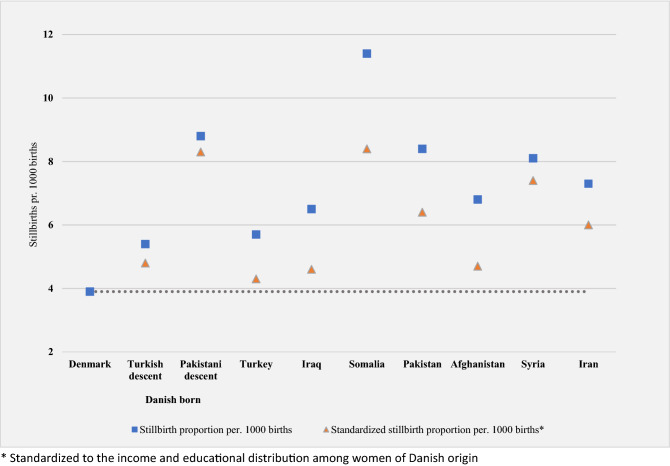
Stillbirth proportions by maternal country of origin in Denmark 2005–2016 and stillbirth proportions standardized by maternal educational level and household income*.

**Figure 2:**
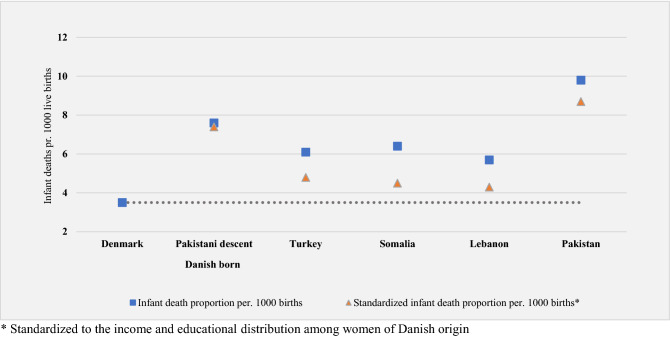
Infant death proportions by maternal country of origin in Denmark 2005–2016 and proportions of infant death standardized by maternal educational level and household income*.

We conducted the standardization analyses in subgroups with a statistically significant increased risk of stillbirth or infant death (Table 2). When standardized to the income and educational distribution among women of Danish origin, the absolute risk estimates were reduced for all country groups for both outcomes (Figs. 1 and 2). For stillbirth, the risk reduction ranged 0.5–3.0 per 1000 births with the lowest reduction among women of Turkish and Pakistani descent and the largest reduction among women from Somalia. For infant death, the risk reduction ranged 0.2–1.9 per 1000 live born with the smallest risk reduction among women with Pakistani descent and origin and the largest among women from Somalia. With a standardized risk estimate of 4.3 stillbirths per 1000 births among women of Turkish origin, the risk was almost reduced to the same level as among women of Danish origin. For all other groups for both outcomes, the standardized risk estimates were still elevated compared to the reference, indicating that low socioeconomic position seems to explain part of the observed ethnic differences in stillbirth and infant death, however, not all and particularly little for the Pakistani minority.

### Sensitivity analyses

The standardization analyses with missing values coded in the lowest and the highest categories of household income and maternal educational level did not change the overall estimates confirming the robustness of the results (see supplementary Tables S1 and S2). Repeating the analyses including births where information about exposure was only recorded in the years following birth did not change the overall results indicating no risk of bias by their exclusion.

## Discussion

We observed considerable disparities in the risk of stillbirth and infant death by maternal country of origin in Denmark in 2005–2016. Women from Pakistan and Somalia were at the highest risk for both outcomes. A notable observation was the persistent risk of stillbirth and infant death among descendants of Turkish and Pakistani immigrants. Low socioeconomic position only partly explained the observed ethnic disparities in stillbirth and infant death.

Our findings of increased risks of stillbirth and infant death among women from Turkey, Pakistan, and Somalia are consistent with the previous Danish research including births from 1981–2003^8^, emphasizing these disparities as an ongoing concern. Direct comparison to the international studies reporting ethnic disparities in stillbirth and infant death is impeded by variation in the definition of immigrants. However, the overall mechanisms leading to poorer reproductive outcomes among immigrant women in Europe are related to the intersection between gender, ethnicity, socioeconomic position, and overall societal power structures^15^. What women want from maternity care has been found to be the same irrespective of country of origin^16^, however from various contexts it has been shown that immigrant women are at increased risk of receiving suboptimal maternity care^17–19^. Miscommunication due to cultural and linguistic barriers has been explained as causes that leads to delays in care with potential fatal consequenses^15^.

Immigrant women from Somalia had the highest observed risk of stillbirth, which is consistent with other studies^7,20^. For this group, delays in care seeking and in health system compliance with management guidelines seem particularly important^21^. Qualitative Scandinavian studies have found that language difficulties, distrust and perceived discrimination, low health literacy, and family and job responsibility act as barriers for attending care for Somali-born and Sub-Saharan African women^22,23^. As reported in other studies^24^, we observed a large frequency of post-term deliveries among women from Somalia. In Denmark, Somali-born women had a high rate of emergency caesarean sections and low rate of planned caesarean sections^25^, indicating gaps in communication and delays in interventions. Interestingly, research indicates that the risk of stillbirth differs with gestational age among women with different origin^5^, which warrants further research.

We have not been able to take the effect of any systematic variations in uptake of prenatal screening for congenital anomalies and for use of terminations into account. A recent statistics show that the use of legal terminations before week 12 is more frequent in women with migrant background^26^, but may use the screening program for congenital anomalies less. These two tendencies may outweigh each other, but we had no opportunity to adjust for any effect of such potential bias.

For women with Pakistani and Turkish origin increased risk of stillbirth and infant death has been linked to congenital anomalies due to consanguinity^27^. Norwegian studies observed attenuated risk estimates of stillbirth and infant death among women with Pakistani origin, when adjusting for consanguinity. However, the risk remained elevated compared to the host population, indicating that consanguinity is only part of the explanation^3,7^. An observed increased risk of gestational diabetes among immigrant women from Pakistan and Turkey in Denmark might likewise play a role for these two groups^28^.

The majority of immigrants with non-Western origin had significantly lower levels of maternal education and household income compared to women of Danish and Western origin. Differences in socioeconomic position explained most of the excess risk of stillbirth among immigrant women from Turkey. For all other ethnic groups, low socioeconomic position could only partly explain the increased risk of stillbirth, however, with varying strength. This minor explanatory strength is in line with other Scandinavian studies^4,7,8^. For infant death, low socioeconomic position explained part of the excess risk among women from Somalia, Turkey, and Lebanon, however, little among women originating from Pakistan.

For the descendants, we can rule out the explanations related to language barriers and newness. To our knowledge, this is the first Danish study examining differences in stillbirth and infant death among descendants. A similar risk of stillbirth and infant death among generations of women with Pakistani descent has been found in Norway^3^. Consanguineous relations were still prevalent, however, less among descendants compared to immigrants^3^. In the UK, the increased risk of stillbirth among immigrants from Pakistan was not persistent in their descendants^29^. In our study, 95% of children born to women of Turkish and Pakistani descent had fathers of non-Danish origin, indicating strong transnational ties. The persistent disparity across generations of immigrants in Denmark cannot be explained by socioeconomy and is a disturbing finding that deserves further investigation, e.g. of discrimination and chronic stress hypothesis.

The population-based register study allows for the inclusion of all live- and stillbirths of women with registered residence in Denmark in the study period^30^. The large sample size enabled inclusion of many categories of maternal origin, despite rare outcomes. Registry information on maternal country of origin is high-quality data^31^. Only 0.3% of the total number of births in 2005–2016 had discordant information about maternal country of origin. We a priori chose to exclude births where information about exposure was only recorded in the years following birth. This due to the assumption that giving birth to a sick or stillborn child might affect the chance of obtaining Danish citizenship or the likelihood of having erroneous records corrected. Repeating the analyses including these births did not change the overall results indicating no risk of bias.

Educational level and household income were indicators of socioeconomic position. They are known risk factors for stillbirth and infant death^10,11^ and capture different mechanisms affecting health^32^. The Danish education registers cover education completed in Denmark and additional self-reported information for immigrants with no Danish schooling records^33^. Immigrants had a large proportion of missing values on educational level^34^. Nevertheless, a number of sensitivity analyses where missing values on maternal education and household income were coded in, respectively, the best off and worst off categories confirmed the robustness of our results.

When standardizing to the income and educational distribution among women of Danish origin it is assumed that these measures have the same meaning irrespective of ethnicity. This assumption can be questioned^32,35,36^. Educational level among immigrants might be less indicative of socioeconomic position, as especially non-Western immigrants with a non-Danish education are more likely to be underemployed according to their educational level^9,32^. A larger proportion of women of Turkish and Pakistani descent were in the highest educational category than immigrant women of same origins. However, this did not transmit to the income of these descendants as more than 70% were in the lowest income category. The same tendency was observed among immigrant women from Iran (Table 1). This might reflect differences in age composition, difficulties in entering the Danish labour market or cultural differences with more families of non-Western origin choosing to have the mother stay-at-home.

The very low level of household income among immigrants from Syria reflects that most Syrians have recently arrived as war refugees. Thus, household income and educational level might not fully capture the concept of socioeconomic position in all groups. Future studies including factors such as length of residency, reason for migration, language proficiency, and employment status could contribute to further elaboration on the influence of socioeconomic position.

This study demonstrates considerable disparity in the risk of stillbirth and infant death by maternal country of origin in Denmark and additionally indicates a noteworthy persistent elevated risk across generations of immigrants. Children of mothers from Pakistan and Somalia were at the highest risk for both outcomes. The interplay with social determinants of fetal and infant mortality explains only part of the increased risk in the largest immigrant groups, and in some cases almost nothing.

## Supplementary Information

Supplementary Information

## Data Availability

All data were accessed and analysed using a remote access connection to Statistics Denmark, were data were anonymized and the non-visibility of individual data secured. Availability of these data is restricted to research institutions with approved license such as the Danish public universities, and so are not publicly available. Access to data can be given upon reasonable request and with permission from Statistics Denmark.
