# Supplementary material for: Social and ethnic disparities in stillbirth and infant death in Denmark, 2005–2016
Source: Sci Rep. 2021 Apr 12;11:8001. doi: 10.1038/s41598-021-87084-3 (PMC8041838; doi:10.1038/s41598-021-87084-3)
Supplement: Supplementary file 1 — Supplementary Information [file 41598_2021_87084_MOESM1_ESM.docx]

**Supplementary tables to the manuscript:** Social and ethnic disparities in stillbirth and infant death in Denmark, 2005-2016

**Authors:** MSc Trine Damsted Rasmussen*, Associate professor Sarah Fredsted Villadsen, Professor Per Kragh Andersen, Associate professor Signe Smith Jervelund, Professor Anne-Marie Nybo Andersen

**Supplementary Table S.1. Crude stillbirth proportions per 1000 births by maternal country of origin in Denmark**

**2005-2016 and stillbirth proportions standardized by maternal educational level and household income***

| **Maternal country of origin** | **Crude stillbirth proportion** | **Standardized stillbirth proportion** | **Standardized stillbirth proportion (worst case)**** | **Standardized stillbirth proportion (best case)***** |
| --- | --- | --- | --- | --- |
| Denmark | 3.9 | - | - | - |
| Turkish descent | 5.4 | 4.8 | 4.7 | 4.7 |
| Pakistani descent | 8.8 | 8.3 | 7.6 | 8.0 |
| Turkey | 5.7 | 4.3 | 4.3 | 4.5 |
| Iraq | 6.5 | 4.6 | 4.9 | 5.1 |
| Somalia | 11.4 | 8.4 | 8.2 | 8.4 |
| Pakistan | 8.4 | 6.4 | 6.5 | 7.1 |
| Afghanistan | 6.8 | 4.7 | 5.0 | 5.2 |
| Syria | 8.1 | 7.4 | 5.5 | 6.5 |
| Iran | 7.3 | 6.0 | 6.3 | 6.6 |

* Standardized to the income and educational distribution among women of Danish origin

** Births with missing information on maternal education level and household income are coded in the worst off categories

*** Births with missing information on maternal education level and household income are coded in the best off categories

**Supplementary Table S.2. Crude infant death proportions per 1000 live births by maternal country of origin in Denmark**

**2005-2016 and infant death proportions standardized by maternal educational level and household income***

| **Maternal country of origin** | **Crude infant death proportions** | **Standardized infant death proportions** | **Standardized infant death proportions (worst case)**** | **Standardized infant death proportions (best case)***** |
| --- | --- | --- | --- | --- |
| Denmark | 3.5 | - | - | - |
| Pakistani descent | 7.6 | 7.4 | 6.4 | 6.7 |
| Turkey | 6.1 | 4.8 | 4.7 | 4.8 |
| Somalia | 6.4 | 4.5 | 4.6 | 4.6 |
| Lebanon | 5.7 | 4.3 | 4.3 | 4.3 |
| Pakistan | 9.8 | 8.7 | 7.5 | 8.2 |

* Standardized to the income and educational distribution among women of Danish origin

** Births with missing information on maternal education level and household income are coded in the worst off categories

*** Births with missing information on maternal education level and household income are coded in the best off categories
